# Quetiapine Shortens the Lifespan of *Caenorhabditis elegans* through DOP-2, DAF-2 and RSKS-1

**DOI:** 10.3390/ijms232112927

**Published:** 2022-10-26

**Authors:** Yizhou Jiang, Uma Gaur, Zhibai Cao, Sheng-Tao Hou, Wenhua Zheng

**Affiliations:** 1 Centre of Reproduction, Development & Aging and Institute of Translation Medicine, Faculty of Health Sciences, University of Macau, Macau 999078, China; 2 Brain Research Centre, Department of Biology, School of Life Science, Southern University of Science and Technology, 1088 Xueyuan Blvd, Nanshan District, Shenzhen 518055, China

**Keywords:** quetiapine, longevity, antipsychotics, *C. elegans*

## Abstract

Recent studies implicate a key role of dopamine signaling in lifespan regulation. Our previous study found that quetiapine, an atypical antipsychotic drug that has antagonistic activity on dopamine D2-like receptors (D2Rs), shortened the lifespan of *Caenorhabditis elegans* (*C. elegans*). However, the detailed mechanism of this effect was not clear. In the present study, we evaluate the effect of quetiapine on aging and explore its underlying molecular mechanism. The results show that quetiapine shortened healthspan in *C. elegans*. The lifespan-shortening effect is dependent on DOP-2, a D2R expressed in worms. Quetiapine shortens lifespan through the *C. elegans* insulin and IGF-1 receptor DAF-2, but not the downstream Akt pathway. Quetiapine-induced lifespan reduction is dependent on RSKS-1, a key protein kinase that functions in mTOR signaling. In addition, the quetiapine effect is also related to mitochondrial function. These findings further support the key role of dopamine signaling in lifespan regulation and promote our insight into the mechanism of action of antipsychotic drugs.

## 1. Introduction

The nervous system plays an active role in lifespan regulation. Drugs targeting neurotransmitter receptors, including antipsychotics and antidepressants, have been shown to affect longevity in *C. elegans* [1,2]. Our recent study showed that the atypical antipsychotic drug quetiapine shortened the lifespan of *C. elegans* [1]. However, the molecular mechanisms underlying the effect were still unclear.

Although they have been clinically used for decades, the mechanisms of action of antipsychotic drugs are still poorly understood. Aripiprazole, a widely used atypical antipsychotic drug, was shown to extend the lifespan of *C. elegans* via signaling through DOP-2, a dopamine D2-like receptor (D2R) expressed in worms [1]. The mechanisms of action of aripiprazole are similar to those of quetiapine, except that quetiapine is a D2R antagonist, whereas aripiprazole possesses an agonistic effect on D2R [3,4,5]. Therefore, it is plausible that the lifespan-shortening effect of quetiapine is also mediated by DOP-2.

While the pharmacological properties of antipsychotics on dopamine and serotonin receptors are relatively clear, little is known about the molecular mechanisms which may account for the side effects of antipsychotic medications [6]. It was reported that antipsychotic drugs could disrupt the normal development of *C. elegans*, but the effect was independent of either dopamine or serotonin signaling [7]. Therefore, it is also possible that the harmful effects of quetiapine on longevity are dependent on additional mechanisms.

In this study, we aim to evaluate the effect of quetiapine on *C. elegans* longevity and its mechanisms. Our results provide critical insight into the mechanism of action of quetiapine and the role of dopamine signaling in regulating longevity.

## 2. Results

### 2.1. Quetiapine Reduces Healthspan but Does Not Affect the Locomotion and Pharyngeal Pumping of C. elegans

**Figure 1:**
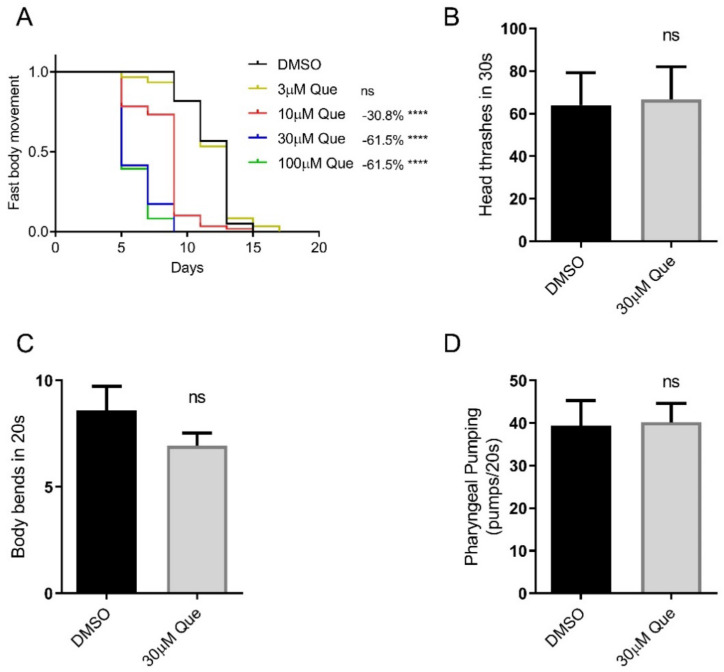
Quetiapine reduces healthspan but does not affect locomotion and pharyngeal pumping of *C. elegans*. (**A**) Fast body movement spans of wild-type (N2) worms cultured at 20  °C on NGM plates containing indicated concentrations of quetiapine (Que) or DMSO (*n* = 60 worms per group) (comparison between DMSO and Que treatment: ns  =  not significant; ****, *p* <  0.0001, log-rank test). (**B**,**C**) Head thrash frequencies (*n* = 15 worms per group) (**B**) and body bend frequencies (*n* = 15 worms per group) (**C**) of N2 animals treated with 30 μM of quetiapine or DMSO for 5 days (comparison between DMSO and Que treatment: ns  =  not significant, *t*-test). (**D**) Pharyngeal pumping rates of N2 animals treated with 30 μM of quetiapine or DMSO for 7 days (*n* = 15 worms per group) (comparison between DMSO and Que treatment: ns  =  not significant, *t*-test). Experiments were performed in at least two independent biological replicates.

In the present study, we first evaluated the effect of quetiapine on healthspan. The results show that quetiapine also dose-dependently shortened the healthspan of *C. elegans*, which is coupled with its effect on worm lifespan (Figure 1A). To further evaluate the effect of quetiapine on health, we tested the head thrash and body bend frequencies of N2 animals treated with 30 μM of quetiapine or DMSO for 5 days. The results show that they were not significantly altered by quetiapine treatment. In addition, we also found that quetiapine treatment did not significantly alter the pharyngeal pumping rate. Taken together, quetiapine reduces healthspan but does not affect the locomotion and pharyngeal pumping of *C. elegans*.

### 2.2. Quetiapine-Mediated Lifespan Shortening Is Dependent on DOP-2

**Figure 2:**
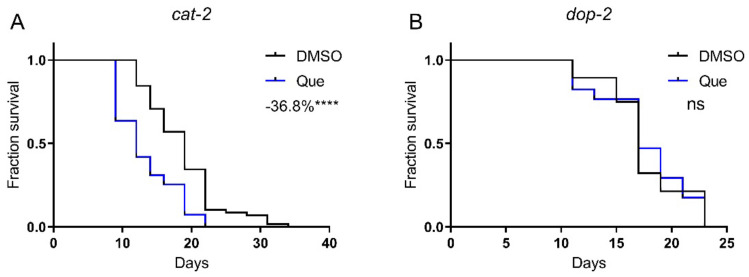
Quetiapine-mediated lifespan shortening is dependent on DOP-2. Survival curves of mutant *cat-2* (*n* = 60 worms per group) (**A**) and *dop-2* (*n* = 30 worms per group) (**B**) treated with 100 μM of quetiapine or DMSO at 20 °C. Data were analyzed by using log-rank test (ns: no significant difference with *p* > 0.05; ****: *p* < 0.0001). Experiments were performed in at least two independent biological replicates.

We then aimed to elucidate the molecular mechanism of the lifespan-shortening effect of quetiapine. Given its antagonistic action on D2R, we first tested quetiapine on the dopamine-deficient mutant *cat-2*, which lacks tyrosine hydroxylase, the rate-limiting enzyme of dopamine synthesis. Strikingly, quetiapine still robustly shortened the lifespan of this mutant, suggesting that its effect seems to be independent of dopamine signaling (Figure 2A). However, we have previously demonstrated that the activation of DOP-2 by aripiprazole extended worm lifespan [1]. Therefore, it is reasonable to speculate that the lifespan-shortening effect of quetiapine is also attributed to its antagonistic effect on DOP-2. In addition, it was reported that the *cat-2* mutant still retained a significant amount of dopamine, indicating that the dependence of dopamine signaling may not be readily revealed by using this mutant [8]. Therefore, we further tested quetiapine on the *dop-2* mutant. As shown in Figure 2B, the mutation on *dop-2* abolished the effect of quetiapine, supporting that it did shorten lifespan through its action on DOP-2.

### 2.3. Quetiapine Shortens Lifespan through DAF-2, but Not the Downstream Akt Pathway

**Figure 3:**
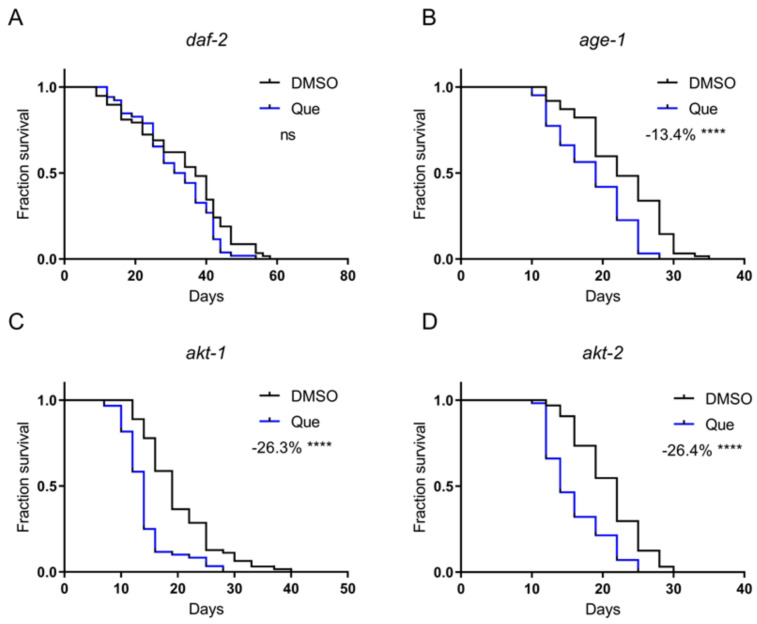
Quetiapine shortens lifespan through DAF-2, but not the downstream Akt pathway. Survival curves of mutant *daf-2* (*n* = 60 worms per group) (**A**), *age-1* (*n* = 60 worms per group) (**B**), *akt-1* (*n* = 60 worms per group) (**C**), and *akt-2* (*n* = 60 worms per group) (**D**) treated with 100 μM of quetiapine or DMSO at 20 °C. Data were analyzed by using log-rank test (ns: no significant difference with *p* > 0.05; ****: *p* < 0.0001). Experiments were performed in at least two independent biological replicates.

Antipsychotic drugs have been shown to activate the insulin/insulin-like growth factor 1 (IGF-1) signaling pathway via DAF-2, the *C. elegans* insulin and IGF-1 receptor [9]. The activity of the insulin/IGF-1 signaling pathway is negatively correlated with lifespan. Therefore, we postulated that quetiapine might mediate lifespan shortening through this pathway. The results showed that quetiapine-mediated lifespan shortening does require DAF-2 (Figure 3A). The classical downstream of DAF-2 are AGE-1 (the *C. elegans* homolog of phosphoinositide 3-kinases) and AKT-1/2 (the *C. elegans* homologs of protein kinase B). We next treated the *age-1*, *akt-1*, and *akt-2* mutants with quetiapine. However, quetiapine still shortened the lifespan of these mutants, indicating that the *C. elegans* Akt pathway was not involved in quetiapine-mediated lifespan shortening (Figure 3B–D).

### 2.4. Quetiapine-Induced Lifespan Shortening Is Independent of the AAK-2–DAF-16 Pathway

**Figure 4:**
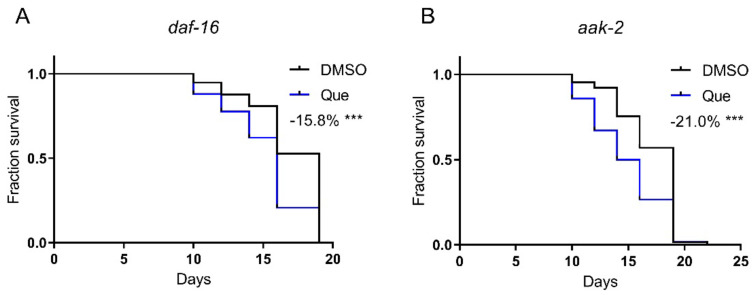
Quetiapine-induced lifespan shortening is independent of the AAK-2-DAF-16 pathway. Survival curves of mutant *daf-16* (*n* = 60 worms per group) (**A**) and *aak-2* (*n* = 60 worms per group) (**B**) treated with 100 μM of quetiapine or DMSO at 20 °C. Data were analyzed by using log-rank test (***: *p* < 0.001).

AAK-2 (the *C. elegans* AMP-activated protein kinase) and DAF-16 (the *C. elegans* FOXO transcription factor) play key roles in regulating lifespan. Our previous study showed that aripiprazole-mediated lifespan extension was dependent on the AAK-2-DAF-16 signaling pathway [1]. Therefore, we hypothesized that quetiapine may also mediate lifespan through this pathway. Nevertheless, quetiapine still shortened the lifespan of the *aak-2* and *daf-16* mutants (Figure 4A,B). These results indicate that quetiapine does not act through the AAK-2-DAF-16 signaling pathway to affect lifespan.

### 2.5. Quetiapine-Induced Lifespan Reduction Is Dependent on mTOR Signaling

**Figure 5:**
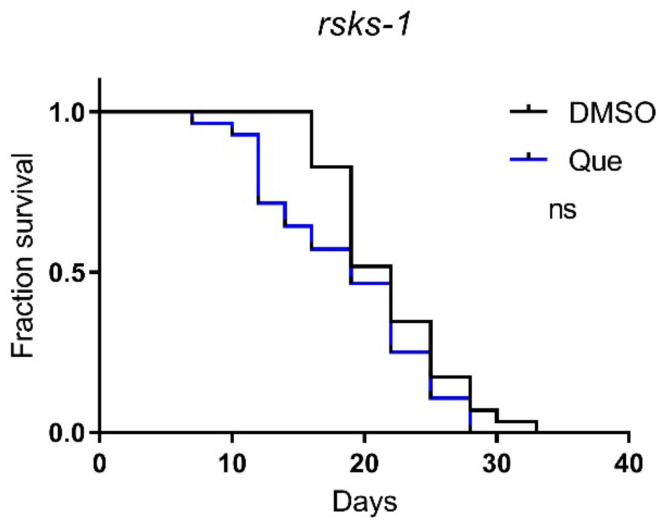
Quetiapine-induced lifespan reduction is dependent on mTOR signaling. Survival curves of mutant *rsks-1* (*n* = 30 worms per group) treated with 100 μM of quetiapine or DMSO at 20 °C. Data were analyzed by using the log-rank test (ns: no significant difference with *p* > 0.05). Experiments were performed in at least two independent biological replicates.

The mTOR signaling pathway plays an important role in lifespan regulation [10]. To further explore the molecular mechanism underlying the effect of quetiapine on lifespan, we tested quetiapine on worms carrying mutations on the *rsks-1* gene, which encodes the *C. elegans* homolog of mammalian ribosomal protein S6 kinase (S6K), a conserved downstream effector of mTOR [11,12]. The results show that the lifespan-shortening effect of quetiapine was abolished in the *rsks-1* mutant (Figure 5), indicating a key role of the mTOR signaling in the effects of quetiapine.

### 2.6. Quetiapine-Induced Lifespan Reduction Is Associated with Mitochondrial Function

**Figure 6:**
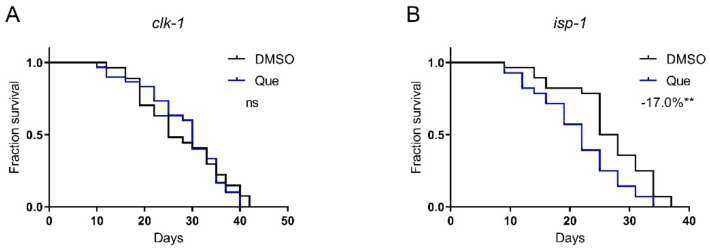
Quetiapine-induced lifespan reduction is associated with mitochondrial function. Survival curves of mutant *clk-1* (*n* = 30 worms per group) (**A**) and *isp-1* (*n* = 30 worms per group) (**B**) treated with 100 μM of quetiapine or DMSO at 20 °C. Data were analyzed by using log-rank test (ns: no significant difference with *p* > 0.05; **: *p* < 0.01).

Aging is accompanied by degenerations in mitochondrial functions, and the damage to mitochondrial proteins reduces lifespan [13,14]. To test whether quetiapine-induced lifespan shortening is associated with mitochondrial function, we tested the drug on the *isp-1* and *clk-1* mutants, which lack the iron–sulfur protein of mitochondrial complex III and a mitochondrial hydroxylase, respectively [15,16]. As shown in Figure 6A,B, quetiapine-mediated lifespan extension was dependent on *clk-1* but not *isp-1*, indicating that it may affect lifespan through a mechanism associated with mitochondrial function.

## 3. Discussion

In this study, we evaluated the effects of quetiapine on the health of *C. elegans*. Besides its effect on lifespan, quetiapine also dose-dependently reduced the fast body movement span; however, the results show that quetiapine did not alter the locomotion and pharyngeal pumping of *C. elegans*. The concentration of quetiapine we used to test these functions was 30 μM, which was shown to be sufficient to induce significant lifespan reduction [1], and was also able to markedly alter the healthspan. In this study, we only measured the head thrash and body bend frequencies on day 5 and the pharyngeal pumping rate on day 7. We did not rule out the possibility that quetiapine may promote the age-associated deterioration of these functions starting at a later age. However, some quetiapine-treated worms started to die from day 7, and the time could be earlier when worms were treated with 100 μM of quetiapine. Therefore, it is difficult to measure these functions at a later stage. In addition, it was reported that D2R signaling itself could regulate locomotor activities. Impaired D2R signaling was shown to cause locomotor impairment, whereas activating D2R could improve locomotor activities [17,18,19]. In the present study, we showed that as a D2R antagonist, quetiapine impaired the fast body movement of *C. elegans*, but did not alter other functions, indicating that the effect of quetiapine on different indicators of locomotion may be selective.

We next investigated whether the quetiapine effect was dependent on its actions on dopamine signaling. We found that it could still shorten the lifespan in *cat-2* mutants. However, since the *cat-2* mutant can still synthesize a significant amount of dopamine, the dependence of dopamine signaling may not be readily revealed by using this mutant [8]. Indeed, we next showed that the lifespan-shortening effect of quetiapine was dependent on DOP-2. Together with our previous reports, we showed that genetic mutation on DOP-2 shortened lifespan. Aripiprazole extends lifespan through its agonistic action on DOP-2, whereas the D2R antagonist quetiapine also shortened lifespan through DOP-2. These results demonstrate a key role of DOP-2 in lifespan regulation. Since quetiapine could also act on serotonin receptors, further studies are needed to test whether the serotonin system is involved in its effect on lifespan.

The mechanisms of action of the antipsychotic drugs are largely unclear, especially for those responsible for their side effects. The “on-target” side effects of antipsychotic drugs include extrapyramidal motor symptoms and excess prolactin release, which are associated with D2R inhibition [20]. Other metabolic side effects, including weight gain, have been considered to be the “off-target” side effects [21]. Our results show that quetiapine has a negative effect on longevity. This effect is mediated by DOP-2, suggesting that its major life-threatening side effects may be attributed to D2R inhibition. In humans, D2Rs are expressed in pancreatic beta cells and mediate insulin secretion [22,23]. In *C. elegans*, we found that both aripiprazole and quetiapine alter lifespan via DAF-2, the worm insulin receptor [1]. In addition, D2Rs were found to regulate dietary restriction responses [1]. These findings suggest that dopamine signaling actively participates in metabolic control, further supporting the notion that adverse metabolic effects of quetiapine may be associated with its action on D2R. Together with our previous finding that activating D2R by aripiprazole has lifespan and metabolic benefits, these results suggest that the drug’s action on D2R may be a prime consideration for minimizing the potential side effects of antipsychotic medications.

Quetiapine-mediated changes in lifespan required DAF-2, which correlates with what we observed in aripiprazole-treated animals [1]. In addition, it was reported that both drugs could activate DAF-2 in *C. elegans* [9]. These findings implicate a key role of DAF-2 in response to antipsychotics treatment. However, neither aripiprazole nor quetiapine regulates lifespan through the Akt pathway, which serves as an important downstream of DAF-2. Therefore, there should be alternative pathways or proteins responsible for the effects of antipsychotics on lifespan. Besides the Akt pathway, another downstream of DAF-2 is HSF-1 (heat shock transcription factor 1), which could be a possible effector of quetiapine-mediated lifespan reduction. The quetiapine effect was also dependent on RSKS-1, a key protein kinase in the mTOR signaling. Conversely, the aripiprazole effect was not related to RSKS-1. It was reported that both insulin/IGF-1 signaling and mTOR signaling could be downstream of each other [10]. In addition, they could also be interconnected via signaling crosstalk involving other proteins or pathways. For example, a common downstream of RSKS-1 and DAF-2 is germline signaling [24], which regulates lipid metabolism and might be associated with the metabolic side effects of quetiapine. DAF-2 and RSKS-1 may function in parallel or in the same pathway in quetiapine-mediated lifespan reduction. These findings suggested that although both aripiprazole and quetiapine target DOP-2 to regulate lifespan, they may elicit both overlapping and distinct downstream metabolic pathways.

To sum up, we found that quetiapine shortened healthspan but did not alter other locomotor activities of *C. elegans*. The lifespan-shortening effect of quetiapine is dependent on DOP-2, DAF-2, and RSKS-1. In addition, quetiapine-mediated lifespan shortening may also be related to mitochondrial pathways. These findings further support the key role of dopamine signaling in lifespan regulation and provide mechanistic insight into the clinical side effects of quetiapine.

## 4. Materials and Methods

### 4.1. Nematode C. Elegans Strains and Their Maintenance

All strains were obtained from the Caenorhabditis Genetics Center (CGC, University of Minnesota) and maintained at appropriate temperature on solid nematode growth medium (NGM) plates seeded with *E. coli* OP50. Strains were maintained at 20 °C unless specified. Strains used in this study are described in Table 1.

### 4.2. Preparation of Reagents

Quetiapine, purchased from Meilunbio (Dalian, China), was dissolved in dimethyl sulfoxide (DMSO) as stock. The drug was added to the liquid NGM before pouring the plates. A final DMSO concentration of 0.1% (*v*/*v*) was maintained under all conditions.

### 4.3. Lifespan Assays

Worms were cultured for three generations without starvation before lifespan assays. Lifespan assays were performed at 20 °C on lifespan assay plates supplemented with 50 μM of 5-fluoro-2′-deoxyuridine (FUdR, Sigma, St. Louis, MO, USA) and seeded with live OP50. The day worms were transferred to lifespan assay plates was set as day 0 for the experiment, and worms were counted every 2–3 days. Animals were scored as dead if they did not move when gently prodded. Worms that crawled off the plate or died from vulva bursting were censored from the experiment.

### 4.4. Phenotype Assays

Fast body movement was measured along with lifespan assays. Worms with continuous sinusoidal movement when responding to tapping the plates were classified as fast body movement. For locomotion assays, worms were raised in the same way as lifespan assays. On day 5, 30 individuals from the control or aripiprazole-treated group were measured for head thrashing and body bend frequencies. Briefly, worms were transferred to fresh NGM plates without bacterial lawn, and body bends in 20 s were recorded. For head thrashing frequency, worms were transferred to a drop of M9 buffer on glass slides and head thrashes in 20 s were measured. For pharyngeal pumping assay, pharyngeal pumps in the 20 s intervals were recorded under a microscope on day 7.

### 4.5. Statistical Analysis

Statistical analyses for all lifespan data were performed using the Kaplan–Meier method and the log-rank test. Head thrashing frequencies, body bend frequencies, and pharyngeal pumping rates were analyzed by unpaired *t*-tests. Error bars were presented as mean ± SEM. The data were analyzed using GraphPad Prism 8 (GraphPad Software, San Diego, CA, USA).

## Figures and Tables

**Table 1:** List of *C. elegans* strains used in this study.

Strain	Genotype
N2	Bristol, wild type
CF1038	*daf-16(mu86) I*
CB1370	*daf-2(e1370) III*
CB4876	*clk-1(e2519) III*
LX702	*dop-2(vs105) V*
MQ887	*isp-1(qm150) IV*
RB1206	*rsks-1(ok1255) III*
RB759	*akt-1(ok525) V*
TG38	*aak-2(gt33) X*
TJ1052	*age-1(hx546) II*
VC204	*akt-2(ok393) X*

## Data Availability

All data generated or analyzed during this study are included in this published article.
